# The Influence of Drivers and Barriers on Urban Adaptation and Mitigation Plans—An Empirical Analysis of European Cities

**DOI:** 10.1371/journal.pone.0135597

**Published:** 2015-08-28

**Authors:** Diana Reckien, Johannes Flacke, Marta Olazabal, Oliver Heidrich

**Affiliations:** 1 Center for Research on Environmental Decisions, Columbia University, New York City, New York, United States of America; 2 Faculty of Geo-Information Science and Earth Observation (ITC), University of Twente, Enschede, The Netherlands; 3 Basque Centre for Climate Change (BC3), Bilbao, Spain; 4 Department of Land Economy, University of Cambridge, Cambridge, United Kingdom; 5 School of Civil Engineering and Geosciences, Newcastle University, Newcastle upon Tyne, United Kingdom; University of Washington, UNITED STATES

## Abstract

Cities are recognised as key players in global adaptation and mitigation efforts because the majority of people live in cities. However, in Europe, which is highly urbanized and one of the most advanced regions in terms of environmental policies, there is considerable diversity in the regional distribution, ambition and scope of climate change responses. This paper explores potential factors contributing to such diversity in 200 large and medium-sized cities across 11 European countries. We statistically investigate institutional, socio-economic, environmental and vulnerability characteristics of cities as potential drivers of or barriers to the development of urban climate change plans. Our results show that factors such as membership of climate networks, population size, GDP per capita and adaptive capacity act as drivers of mitigation and adaptation plans. By contrast, factors such as the unemployment rate, warmer summers, proximity to the coast and projected exposure to future climate impacts act as barriers. We see that, overall, it is predominantly large and prosperous cities that engage in climate planning, while vulnerable cities and those at risk of severe climate impacts in the future are less active. Our analysis suggests that climate change planning in European cities is not proactive, i.e. not significantly influenced by anticipated future impacts. Instead, we found that the current adaptive capacity of a city significantly relates to climate planning. Along with the need to further explore these relations, we see a need for more economic and institutional support for smaller and less resourceful cities and those at high risk from climate change impacts in the future.

## Introduction

Cities are seen as important agents for the implementation of climate change mitigation and adaptation actions, as they have less complex governance structures than national and international bodies, while at the same time sufficient political power to implement actions [1,2]. Moreover, in highly urbanized regions, adaptation and mitigation actions will have to take place in or with cities. However, a recent analysis of urban climate change responses in Europe—a highly urbanized region perceived as being a leader in terms of environmental policies [3]—showed that 35% of large and medium-sized cities have no dedicated mitigation plan, 72% have no adaptation plan, and only 25% have both an adaptation and a mitigation plan with quantitative targets for greenhouse gas (GHG) emission reductions (see S1 Table). Moreover, there is considerable diversity within as well as across European countries, both in terms of availability of climate change plans and the ambitions of GHG reduction targets [4]. How can such diversity be explained and what drives cities to plan to mitigate and adapt to climate change or prevents them from doing so? It is the aim of this paper to test the association between urban characteristics and the development of urban climate change plans, and to identify common factors that influence urban responses to climate change.

Uncertainty about climate change impacts is often claimed to be an obstacle to the implementation of climate actions. However, our scientific understanding to date shows that improved quantity and quality of climate information and greater certainty do not necessarily lead to more and better (i.e., locally adapted and appropriate) climate change actions [5–7]. A number of obstacles to the implementation of climate change actions are at play [8]. Both, the fourth and fifth assessment reports of the Intergovernmental Panel on Climate Change (IPCC AR4 and AR5) recognize the influence of drivers and barriers, or opportunities and constraints, on adaptation and mitigation efforts in cities and human settlements [9–11].

Barriers are defined as “obstacles that can be overcome with concerted effort, creative management, change of thinking, prioritization, and related shifts in resources, land uses, [and] institutions” ([8], p. 22027). Overcoming obstacles also requires sufficient political will, social support, resources, and effort [12]. A barrier is therefore a hindrance that can be overcome, and is therefore not insurmountable. Barriers are distinguished from limits, which are absolute and unsurpassable [13]. Some authors regard biophysical and locational factors as limits (to adaptation)[14]; we include environmental and locational factors as barriers in a wider sense, as we assume that a certain degree of adaptation is possible to most of the locational factors experienced in Europe. Nonetheless, barriers to adaptation are mostly, “social factors and conditions (that) hamper our ability to adapt proactively to future environmental changes” ([15], p.119), including cultural and cognitive barriers arising from different perceptions of vulnerability, adaptive capacity and risk [16–19]. Drivers may be seen as the opposite of barriers and as stimulators of political will, social support, resources and efforts. They can range from socio-economic macro-variables, such as population and economic growth, to environmental patterns and public opinion [20]. In the context of climate change policies, drivers are understood as activities, processes or patterns that produce positive incentives for climate action (adapted from OECD; see [21]).

However, there is danger that using the terms ‘barriers’ and ‘drivers’ may suggest a clear-cut causality between factors such as social conditions, natural processes and physical patterns, and their impacts on adaptation and mitigation efforts. Such clear-cut causality may not always exist, as drivers and barriers can be context specific. We acknowledge that barriers and drivers do not imply mono-causality and that their degree of influence or explanatory power with respect to climate change response may to some degree vary across contexts [22]. However, we assume that a number of drivers and barriers are common across contexts and scales (see, e.g. the IPCC’s Fifth Assessment Report (AR5) [23], [24]).

Over the last decade, there has been a substantial increase in studies of the drivers and barriers that influence mitigation and adaptation efforts. A recent literature review [15] found that barriers differ across governance levels, i.e. cities versus nation states, and across domains, i.e. mitigation versus adaptation. It was also shown that the vast majority of scientific papers on barriers to adaptation (N = 81) focus on the local or regional level and are based on a small sample size or single case studies. There have been very few comparative studies of barriers and drivers affecting climate change responses across countries, scales, and domains, and generalized knowledge about common influence factors is extremely limited [15]. This may be related to the shortage of comparable data for large samples of urban areas and in turn to problems of definition (for example of ‘urban’ or ‘resident population’), and measurement [22]. However, to provide generalized knowledge beyond the level of individual case-studies, broad-scale studies are needed.

Studies have identified a range of factors that influence climate change mitigation and adaptation efforts [25–29]. According to Bulkeley et al. ([22], p. 13), the factors most strongly identified with mitigation planning are: leadership, competencies, resources, and political economies. The IPCC highlighted institutional, legal, financial and cultural barriers, as well as administrative and political ones as an obstacle to mitigation [24]. For adaptation, the IPCC identified changes in population, age structure, income, technology, relative prices, lifestyle, regulation, and governance as important [23]. However, these socio-economic drivers or barriers to adaptation are not well understood [23,30]. Additional factors influencing climate change adaptation include: scientific information and knowledge [18,31], local governance capacity, multilevel governance, networks and partnerships, community engagement, and education ([22], p.33). McEvoy et al. ([32], p.188) identified the main climate-related hazards of concern (in the UK) as being increased temperature, changing precipitation patterns and an increase in the frequency of extreme events, signifying that environmental factors can also be important. Overall, the influence factors of adaptation seem less clear. The capacity to adapt is believed to be more context and site specific [30,33].

Clearly, there are many factors that could be considered, though institutional, socio-economic and environmental factors figure prominently in several lists. We therefore narrow down our research question and review the institutional, socio-economic, and environmental factors that potentially influence local climate change planning. We select a number of site-specific factors found in the literature and statistically test their relation to climate change responses—urban mitigation and adaptation plans—across broader scales, i.e. in 200 large and medium size cities that are regionally representative of 11 European countries investigated.

## Review of Factors that Can Act as Climate Change Drivers and Barriers

### Institutional factors

Institutions are seen as crucial for urban climate change response. They provide support for implementing both adaptation and mitigation efforts [34]. For mitigation, both the political will and the institutional capacity to implement mitigation plans are seen as important [11], as are financial and technical assistance, and know-how [11]. Successful adaptation seems to depend on the establishment of a shared science-policy competence [26,35,36], that facilitates access to climate change information, impact analysis and interpretation [37]. Strong leadership is another important factor [23,38]. If leadership is weak, identifying and agreeing goals and criteria can become a barrier [8] and undermine the capacity and willingness to make decisions [5,18].

Climate networks foster knowledge and information exchange [39], establish norms and, most importantly, give access to financial and political resources [25,40–42]. However, climate networks are “not as open or inclusive as some of the more optimistic interpretations would lead us to believe” ([43], p.537), as hierarchies of involvement can lead to the marginalization of some actors. Relatively little is known about the participation of cities or city leaders in climate networks other than the big ones—i.e. C40 [44] and Cities for Climate Protection (CCP)[40]. In particular there is little information about the influence of climate networks in Europe, as most publications concentrate on the CCP in the US [40,41,45].

Higher-level, i.e. national, sub-national or regional government support, is also frequently mentioned as a driver of climate action, both for mitigation (see [1] and [36] for the USA, and [46] for UK cities), and adaptation (see [27] for Australia, [47] for Germany, [48] for Latin-America, and [49] for Sweden). However, higher-level government support is not always sufficient to drive urban climate plans. De Gregorio Hurtado et al. [50] show that Italian cities develop more climate change plans than Spanish cities, although Spain has a national climate strategy and Italy does not. To make a national strategy effective for translation onto the local level, detailed and clear information on how to use the national strategy and its implications for the regional and local level is needed. According to these authors, this is currently lacking in Spain.

### Socio-economic factors

Socio-economic factors have been identified as important drivers of local macro-trends [51], such as CO_2_ emissions [28]. However, less is known about the influence of socio-economic factors on local climate change planning and policy.

For mitigation, higher personal incomes (affluence) are associated with increased household GHG emissions [24], thereby potentially impeding mitigation [52], and high public costs and the lack of public financial resources are often mentioned as barriers [18,24,49]. However, incomes and public costs play an ambiguous role in adaptation, and the policy and planning contexts are of extreme importance. For example, while high public costs are an impediment to local adaptation in Norway [49] and Australia [27], the financial capacity of a city does not seem to influence adaptation efforts in the US [53]. In the US, aggregated personal incomes and poverty rates are more important influences on adaption than public budgets, possibly reflecting the country’s distinctive planning systems and practices.

Poverty rates, together with factors such as age, gender, ethnic minority status, and education, are also frequently mentioned in relation to social vulnerability to potential climate change impacts [54,55]. Assuming that the experience of climate change impacts can shape action [29,56,57], the presence of a large percentage of vulnerable groups in a city might be linked to increased adaptation planning efforts [23]. Vulnerability is a strong determinant of current and future climate change risk. Highly vulnerable cities (and populations) should therefore particularly invest in adaptation.

Investments in urban performance and change increasingly involve modern Information and Communication Technologies (ICT), as for example in the "smart city" concept [58]. Advocates of smart cities maintain that the use of modern (information) technology will allow European cities to achieve sustainable urban development more easily [58]. If so, it seems reasonable to assume that smart cities—and similar initiatives—are also forerunners in climate change planning.

There are potentially a multitude of socio-economic factors that drive climate change planning, including many that cannot be considered by this study. Though a larger set of factors may potentially better explain differences and commonalities between mitigation and adaptation or among regions (which Kriegler et al. [59] refer to as shared socio-economic pathways), there is no commonly agreed set of socio-economic factors for mitigation and adaptation analysis.

### Environmental factors

Studies that investigate the relation between mitigation planning and urban environmental factors are rare, although geographical and locational factors are noted to influence the belief in climate change and willingness to mitigate [60] and a number of locational factors are related to GHG emissions. For example, lower temperatures in January entail higher emissions associated with home heating, while warmer summers result in higher electricity consumption associated with space cooling [61]. However, urban environmental and locational factors have been found to be associated with membership of climate change mitigation networks. In the US, for example, cities in areas at high risk from climate change (e.g. measured as expected temperature change, casualties of extreme weather events, and coastal proximity) are more likely to be members of the Cities for Climate Protection (CCP) program. Results also show that the odds of a locality joining the CCP can be predicted from the surrounding landscape characteristics [41].

Environmental factors acting as barriers or drivers of climate change adaptation are related to climate change impact, risk and vulnerability. Vulnerability is conceptualized as a function of weather and climate exposure, sensitivity to weather and climate (also referred to as climate change impact) and adaptive capacity to react to the impact [62]. Vulnerability describes the degree of past, current or future susceptibility of people or systems to climate change. Studies show that the temporal dimension is very important for the perception of exposure to climate change and thus for climate change planning. For example, a case study of Norwegian communities [29] found that adaptation efforts are mainly driven by past experiences of weather extremes and not by anticipated future impacts of climate change. This finding is supported by a study of the relationship between adaptation and flood risk versus flood damage in the US [53]. It was found that flood risk and flood damage significantly drive adaptation, but that experience of impacts and flood damage is a more powerful predictor of adaptation than anticipated impacts.

### Composite vulnerability factors

We accept that determining and measuring factors that influence climate change vulnerability and adaptive capacity is challenging, and the subject of debate among scholars [63]. Nevertheless, a city is potentially exposed to multiple climate risks as well as a multitude of other stressors now and in the future [64], which in combination influence the severity of climate change impacts as well as the capacity to respond to climate-related events [65]. This conceptualization is at the heart of the vulnerability concept, as it includes institutional, socio-economic and environmental factors [66]. Likewise, drivers of climate change planning potentially comprise a range of factors from these three domains [30], which may be captured in aggregated vulnerability indices that combine a number of factors from different domains into one measurement. These are used when the multidimensional nature of the concept under investigation cannot be captured using only one factor [67]. We test the influence on climate change planning of aggregated future climate change impacts (from different climate change stressors), aggregated current adaptive capacity, and aggregated future vulnerability [68].

## Methods

We use a representative sample of 200 large and medium-sized cities from 11 European countries (S1 and S2 Tables). The cities are among those selected for the European Commission’s Urban Audit (UA)[69]. The sample represents all UA cities from the 11 countries where authors are familiar with the language and the country’s urban and climate policies [4]. The sample of cities accounts for 16.8% of the EU-27 population (2008); the total population of the 11 countries represents 72.1% of the EU-27 population (2008).

For this sample of cities, we developed a database containing a number of city characteristics, as well as information on the existence and content of urban adaptation and mitigation plans—which are the indicators used to assess climate change response [4]. We consider all strategic policy and planning documents that contain the words ‘climate change’ in the title or refer to climate change in the introduction, e.g. as a motivation for the plan's development (see S1 Text for the selection process of planning/ policy documents). Thus climate change and sustainable energy plans are included, but other sectoral plans, such as transport, waste management or flood protection plans are excluded. Although the latter might be relevant for climate change, their motivation and central aim are largely different. Adoption of the plan in question is not a necessary condition for inclusion if a draft document or sufficient information about the plan and its content is publicly available. A full list of the analysed climate change plans are given in S2 Table.

The existence of a draft, approved and/or published climate change adaptation and mitigation plan, as defined using the criteria mentioned above, are the dependent variables. Based on an inductive approach for indicator development as described by Hinkel [63], a number of institutional, socio-economic, and environmental characteristics of cities are identified from the literature as independent factors, forming potential barriers to, or drivers of climate plan development (Table 1). We test the associations with 30 independent factors, some at multiple points in time (S2 Text). The selection is to some extent contingent on the availability of data, which is often limited for urban areas, compared for instance with national data. Moreover, not all factors found in the literature are easily transferable, quantifiable or measurable, or available in official statistics for all cities studied. We therefore also use appropriate proxy variables that are available for all cities, such as climate network membership for information exchange. Data are obtained from Eurostat [70], which provides data relating to a number of urban characteristics as part of the EU-wide Urban Audit; membership websites of climate networks; and the ESPON Climate project [68], which calculated aggregated indices of climate change impacts, adaptive capacity and vulnerability for European regions (S2 Text provides detailed information on factors, metrics, year, etc.).

**Table 1:** Potential drivers and barriers reported in the literature and factors tested in this study.

		Institutional factors	Socio-economic factors	Environmental factors	Composite vulnerability factors
**Factors reported in the literature**	Mitigation factors	• Climate networks Access to financial and technical assistance	•	• Winter and summer temperatures	•
	Adaptation factors	• Shared science policy-competence	• Social vulnerability: e.g. age, gender Aggregated individual income, high poverty rates	• Flood risk Flood damage	• Anticipated climate impacts
	Joint mitigation and adaptation factors	• Higher level, e.g. national support, guidance or decree Political leadership and political will Scientific knowledge and information exchange	• Individual incomes Costs/ financial capacity of municipalities Use of modern information technology and quality of knowledge communication	• Coastal proximity Exposure to weather extremes	• Experienced impacts
**Factors tested in this study**		• Adoption of national climate change strategies Member of Climate Alliance Member of C40 member Member of Covenant of Mayors Covenant of Mayors: Plan submitted Member of ICLEI	• Population age Population size Population density Gross Domestic Product (GDP) per capita Unemployment rate Smart city index	• Proximity to coast Low elevation coastal zone Altitude above sea leve lHours of sunshine Average temperature of warmest month Average temperature of coldest month Number of rainy days Total amount of rainfall Proportion of green space Availability of green space	• Aggregated Impact Aggregated Vulnerability Combined Adaptive Capacity Combined Mitigative Capacity

The ESPON Climate project defines climate change impact as a weighted combination of potential physical, environmental, social, economic and cultural impacts of climate change in the period between 2071–2100 (as compared to 1961–1990, IPCC SRES A1B scenarios). Weights are based on results of a Delphi survey by the ESPON Monitoring Committee. The impact is calculated from regional data as a combination of different forms of exposure to climatic changes (such as change in mean temperature, mean precipitation, number of summer days, heavy precipitation, and snow days, etc., plus river and coastal flooding resulting from a one meter sea level rise) and recent data on physical, social, economic, environmental and cultural sensitivity (such as sensitivity of transport infrastructure and tourism to climate, of populations to floods, of forests to fire, of soils to erosion, and of Natura 2000 and World heritage sites to climate). Adaptive capacity is calculated as a weighted combination of economic capacity, infrastructure capacity, technological capacity, knowledge and awareness, and institutional capacity [68,71]. Vulnerability is calculated as a combination of potential regional impacts of climate change and regional capacity to adapt to climate change. Mitigative capacity is calculated as a function of awareness, ability and action, measured using indicators of integration of climate change into educational programs, attitudes to climate change, availability of technology and non-carbon energy sources, government effectiveness, income per capita, age dependency, and unemployment, among others.

The statistical analysis involves correlation and regression analyses ([53,72,73], see for similar analyses in other contexts). Non-parametric correlation tests are carried out with Kendall's τ. Factors correlating highly significantly (p<0.01) with either a mitigation or an adaptation plan, but not too strongly among each other, are included in binary logistic regressions (forced entry method). Diagnostic statistics (Cook's distance, the leverage statistic, standardized residuals and DFBeta values) are used to test undue influences on the model and to detect outliers. Tests for the linearity between continuous predictors and the logit of the dependent variable as well as multicollinearity are included.

## Results and Discussion

**Fig 1:**
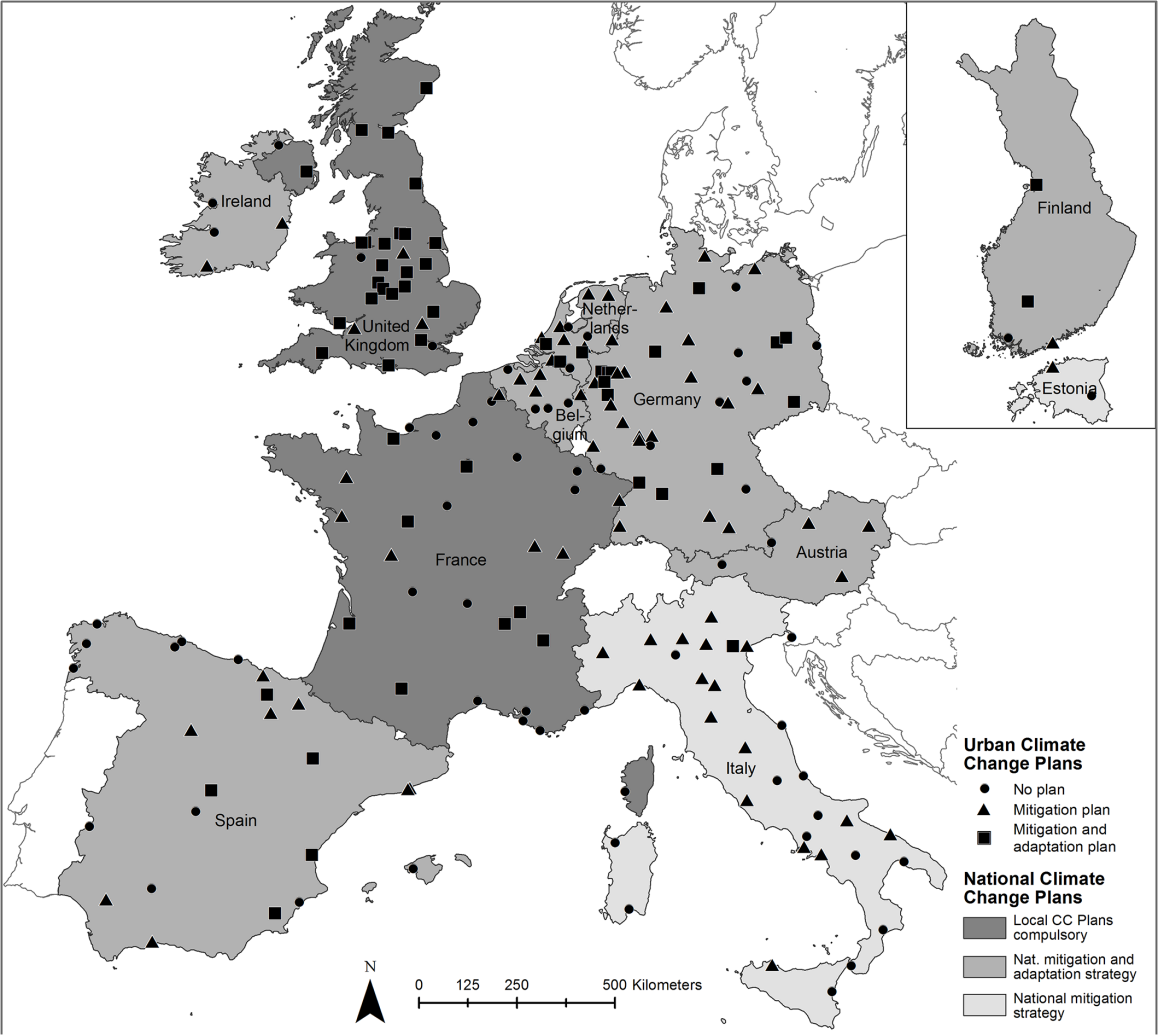
Distribution of climate change adaptation and mitigation plans across European cities, their respective national strategies. Fig 1 shows the location and distribution of cities and countries contained in the analysis. Cities are equally distributed within each country [69]. The sample covers a wide range of countries and climate regions across Europe. Pictograms indicate the location of cities surveyed and the existence of an urban mitigation plan or a mitigation and adaptation plan, if any (there was no city with an adaptation plan, only). Key: CC–climate change; nat.–national.

Fig 1 shows the distribution of climate change adaptation and mitigation plans across European cities, as well as of the corresponding national strategies. The existence of urban climate change plans can be visually compared with the existence and strength of national strategies and directives, shown by the grey colour shading of countries. The United Kingdom—where local climate change plans are compulsory—is an example of a country where urban climate change plans are almost universal. The abundance of climate plans in Britain is due to special circumstances: there is a legal obligation, i.e. the Climate Change Act 2008, on every municipality to have a climate change plan in place. As part of this national legislation British cities are required to plan for projected climate change impacts, and to take action to enhance the cities’ adaptation and mitigation potential. Similar legislation in other European countries exists only in France, which has recently adopted a similar scheme as part of Le Grenelle de l’Environnement, making Territorial Climate and Energy Plans (Plans Climat-Energie Territoriaux or TCEPs) legally mandatory. However, as the example of France shows (see Fig 1), even when legislation requires the development of urban climate change plans these can take a while to appear. By the end of 2012, by which time all major French cities were required to have both a mitigation and an adaptation plan, 14 French cities out of the 35 in our sample still lacked any plan. Most other European countries published national climate change strategies without binding plans for cities. In the countries with national adaptation and mitigation strategies but no specific directive for cities, such as Germany and Spain, quite a large number of cities have climate change mitigation plans, fewer have both an adaptation and mitigation plan, and none has an adaptation plan only. Fig 1 shows that having a national, but for cities non-binding climate change strategy does not guarantee the development of urban climate change plans, although national plans presumably facilitate urban plans by acting as guidance documents. However, our sample shows that this guidance potential is not always taken advantage of.

The existence of plans differs markedly across countries. For example, almost all British cities have a climate plan in place (93% have a mitigation plan, 80% an adaptation plan), while Spanish (50% and 19%) and Italian cities (56% and 3%) are generally less active (S1 Table). Across the sample, 35% of the cities have no dedicated mitigation plan, 72% have no adaptation plan, and only 25% have both an adaptation and a mitigation plan with quantitative targets for greenhouse gas (GHG) emission reductions. Overall, there are fewer adaptation plans than mitigation plans in European cities, despite the widely acknowledged leadership role of Europe in terms of environmental policy and governance and the availability of basic governance functions and infrastructure required for urban climate change planning ([22], see p.78). Some of the studied plans appear tokenistic and adaptation plans are generally less concrete than mitigation strategies (for example: calling for more scientific studies, urban greening or better cooperation among urban stakeholders). However, general recommendations of this nature can still form the basis for good quality plans as long as the role and scope of climate strategies within the wider planning process are clearly defined [22,46]. The EC’s recently (March, 2014) launched Covenant of Mayors initiative for adaptation to climate change (http://mayors-adapt.eu/; access: 20^th^ March 2015) is a demonstration of institutional awareness of the paucity of adaptation guidelines available to local authorities. Following up the network’s activities and outputs would be very useful for future research.

It is outside the scope of this analysis to determine whether the drivers of climate change plans on the national level differ from those at the urban level. For example, projected high impacts of climate change may act as a driver for the development of national plans, as much as they might accelerate the development of urban climate change plans. However, for the purposes of analysis, it is desirable to compare cities where the legal context is similar. For this reason, the following analysis excludes the British cities.

### Correlation analysis

Correlation analysis yields associations between climate change plans and potential drivers and barriers affecting the development of plans for a large sample of European cities. We determine general, broad-scale influences on the development of climate change plans rather than location and context-specific factors. Within the list of tested factors 13 out of 16 factors were significantly (*p*>0.05) related to mitigation (8 drivers, 5 barriers) and 13 out of 16 factors were significantly related to adaptation (6 drivers, 7 barriers). Table 2 shows the significant factors and most recent data point available. The full table with all factors and multiple years is available in S3 Table.

**Table 2:** Drivers and barriers of climate change adaptation and mitigation plans across European cities. Correlations are one-tailed. Data in **bold** highlight factors that are significant on the *p* < 0.05 level. Data in *italic* denote the exact *p* value. Details to factors, units, time dimension and sources are given in S2 Text. The full list of tested factors, including non-significant relations are provided as S3 Table. M plan–Mitigation Plan; A Plan–Adaptation Plan; CoM–Covenant of Mayors; GDP–Gross Domestic Product; LECZ–Low Elevation Coastal Zone; T–Temperature; CC–Climate Change; AC–Adaptive Capacity; Vuln.–Vulnerability.

				1	2	3	4	5	6	7	8	9	10	11	12	13	14	15	16	17	18
CC Plan	1	M Plan	*r*																		
			*p*	.																	
	2	A Plan	*r*	**0.393**																	
			*p*	*0*.*000*	.																
Institutional factors																					
	3	CoM member	*r*	**0.380**	**0.290**																
			*p*	*0*.*000*	*0*.*000*	.															
	4	Climate Alliance	*r*	**0.344**	0.106	-0.080															
			*p*	*0*.*000*	*0*.*084*	*0*.*148*	.														
	5	C40 member	*r*	**0.181**	**0.177**	**0.217**	-0.075														
			*p*	*0*.*009*	*0*.*011*	*0*.*002*	*0*.*164*	.													
	6	ICLEI member	*r*	**0.188**	**0.156**	**0.247**	0.034	**0.293**													
			*p*	*0*.*007*	*0*.*022*	*0*.*001*	*0*.*331*	*0*.*000*	.												
Socio-economic factors																					
	7	Population size	*r*	**0.377**	**0.203**	**0.395**	**0.205**	**0.290**	**0.166**												
			*p*	*0*.*000*	*0*.*001*	*0*.*000*	*0*.*001*	*0*.*000*	*0*.*005*	.											
	8	Population density	*r*	**0.183**	**0.116**	**0.294**	**-0.138**	**0.206**	**0.128**	**0.286**											
			*p*	*0*.*004*	*0*.*047*	*0*.*000*	*0*.*023*	*0*.*001*	*0*.*032*	*0*.*000*	.										
	9	GDP/ head	*r*	**0.376**	**0.139**	**0.170**	**0.407**	**0.164**	**0.199**	**0.282**	0.093										
			*p*	*0*.*000*	*0*.*014*	*0*.*004*	*0*.*000*	*0*.*005*	*0*.*001*	*0*.*000*	*0*.*052*	.									
	10	Unemployment rate	*r*	**-0.298**	-0.103	-0.104	-0.095	-0.099	-0.068	**-0.116**	0.099	**-0.387**									
			*p*	*0*.*000*	*0*.*086*	*0*.*083*	*0*.*105*	*0*.*095*	*0*.*184*	*0*.*031*	*0*.*071*	*0*.*000*	.								
	11	Smart Cities rank	*r*	-0.021	**-0.330**	-0.194	0.034	*/*	**-0.298**	-0.093	-0.210	-0.156	0.074								
			*p*	*0*.*443*	*0*.*012*	*0*.*092*	*0*.*409*	*/*	*0*.*021*	*0*.*228*	*0*.*092*	*0*.*102*	*0*.*316*	.							
Environmental locational factors																					
	12	LECZ	*r*	-0.096	**-0.156**	0.107	**-0.223**	0.016	0.022	0.092	**0.143**	**-0.111**	0.075	-0.024							
			*p*	*0*.*105*	*0*.*021*	*0*.*082*	*0*.*002*	*0*.*418*	*0*.*388*	*0*.*075*	*0*.*019*	*0*.*039*	*0*.*161*	*0*.*436*	.						
	13	Coastal proximity	*r*	**-0.170**	**-0.196**	0.070	**-0.219**	0.008	-0.016	**0.134**	0.104	**-0.152**	-0.013	0.044	**0.712**						
			*p*	*0*.*014*	*0*.*005*	*0*.*181*	*0*.*002*	*0*.*460*	*0*.*418*	*0*.*018*	*0*.*066*	*0*.*008*	*0*.*431*	*0*.*382*	*0*.*000*	.					
	14	Summer T	*r*	**-0.110**	**-0.122**	0.074	**-0.258**	0.080	**-0.150**	0.062	-0.012	**-0.256**	**0.132**	**0.303**	0.093	**0.135**					
			*p*	*0*.*048*	*0*.*032*	*0*.*130*	*0*.*000*	*0*.*114*	*0*.*012*	*0*.*131*	*0*.*421*	*0*.*000*	*0*.*023*	*0*.*008*	*0*.*080*	*0*.*020*	.				
	15	Winter T	*r*	**-0.154**	0.008	0.069	**-0.355**	0.044	**-0.112**	**0.095**	**0.178**	**-0.234**	0.105	**0.220**	**0.287**	**0.286**	**0.184**				
			*p*	*0*.*010*	*0*.*453*	*0*.*147*	*0*.*000*	*0*.*251*	*0*.*045*	*0*.*041*	*0*.*001*	*0*.*000*	*0*.*055*	*0*.*040*	*0*.*000*	*0*.*000*	*0*.*000*	.			
Composite vulnerability factors																					
	16	Future CC impact	*r*	-0.087	**-0.167**	0.081	**-0.319**	**0.108**	-0.027	0.084	0.052	**-0.087**	**-0.106**	**0.239**	**0.307**	**0.192**	**0.361**	**0.367**			
			*p*	*0*.*087*	*0*.*005*	*0*.*102*	*0*.*000*	*0*.*045*	*0*.*338*	*0*.*057*	*0*.*186*	*0*.*049*	*0*.*047*	*0*.*025*	*0*.*000*	*0*.*001*	*0*.*000*	*0*.*000*	.		
	17	Current AC	*r*	**0.240**	**0.225**	0.070	**0.305**	0.035	**0.204**	**0.100**	**0.120**	**0.440**	**-0.221**	**-0.735**	**-0.154**	**-0.231**	**-0.470**	**-0.285**	**-0.275**		
			*p*	*0*.*000*	*0*.*000*	*0*.*135*	*0*.*000*	*0*.*292*	*0*.*001*	*0*.*028*	*0*.*017*	*0*.*000*	*0*.*000*	*0*.*000*	*0*.*007*	*0*.*000*	*0*.*000*	*0*.*000*	*0*.*000*	.	
	18	Future CC vuln.	*r*	**-0.142**	**-0.170**	0.086	**-0.398**	**0.116**	-0.032	0.023	-0.039	**-0.276**	0.045	**0.310**	**0.330**	**0.324**	**0.437**	**0.352**	**0.710**	**-0.548**	
			*p*	*0*.*015*	*0*.*005*	*0*.*094*	*0*.*000*	*0*.*038*	*0*.*312*	*0*.*339*	*0*.*255*	*0*.*000*	*0*.*246*	*0*.*008*	*0*.*000*	*0*.*000*	*0*.*000*	*0*.*000*	*0*.*000*	*0*.*000*	.
			*N*	170	170	170	170	170	170	165	141	169	120	33	170	170	156	156	165	170	157

For mitigation, institutional and socio-economic factors are the most important influences, with Covenant of Mayors (CoM) membership, population size, GDP per capita and Climate Alliance membership being the four most influential. All these factors are drivers of mitigation plans with high certainty, i.e. with positive correlation coefficients and *p*> 0.0001. These results show that large and prosperous cities are disproportionally represented among the centers of climate change planning [74,75]. Larger cities also engage much more often in climate networks, and are able to harness their services. Like CoM, membership of the C40 group and ICLEI are also significant drivers, but with less influence and lower certainty. This suggests that climate networks, which apart from ICLEI were mostly founded to support mitigation, are relatively successful. Another driver with high certainty is current adaptive capacity, which is a composite index based on an assessment of technological, informational, economic, institutional and infrastructural factors.

A barrier to the development of urban mitigation plans is—with high certainty—the unemployment rate of a city. This suggests that mitigation planning might be viewed as an impediment to fighting unemployment and therefore as being achievable only for cities with a low unemployment rate. Mitigation planning is surely not used to raise employment, but more likely viewed as requiring commitment of resources that are urgently needed for other uses, e.g. for investment in jobs. Other significant barriers include locational and environmental factors, such as close proximity to the coast, high summer and winter temperatures, and future vulnerability—although the latter with somewhat lower explanatory power and significance level. Future risk does not seem to motivate cities to mitigate GHG emissions today. The results of our pan-European study therefore contrast with those from New Zealand, where it was found that proximity to the coast increases belief in climate change and support for mitigation policies [60]—although the belief in climate change and the acceptance of policies are slightly different from enacting climate change plans or implementing actions. Our results suggest that climate risk is not an effective driver of climate action [29,32], maybe due to what has been called the psychological barrier, i.e. a feeling that “climate change (risk) is too uncertain, and likely to happen in distant places and times, to people unlike oneself“([60], p.1). It is noteworthy that risk is not an effective driver of climate change action even in high risk areas.

Not significant—at least on the larger European level—are the following factors: the smart city index, median age of the population, location in the low elevation coastal zone or high above sea level, sunshine hours per day or rain days per annum, amount of rainfall, proportion of green space and access to it, or predicted future climate change impacts and current mitigative capacity (documented in S3 Table). This means that smart cities do not combine ICT implementation processes with climate change mitigation plans. It is also noteworthy that cities in the low elevation coastal zone, in mountainous areas, and cities with large amounts of rainfall and/or lots of rainy days do not invest in mitigation more than other cities. Astonishingly, nor do very ‘green’ cities engage more in mitigation than others. This means that urban greenery and access to green space does not necessarily relate to mitigation planning, that could be considered another form of ‘environmentally friendly’ behaviour.

We would expect to see a degree of overlap between factors affecting adaptation and mitigation and, indeed, some drivers of mitigation are also drivers of adaptation planning. Among these, CoM membership, the population size of the city, and current adaptive capacity are the most influential and significant (*p*>0.001) drivers of adaptation. C40 and ICLEI membership, as well as average GDP per capita and population density are also associated with adaptation planning, but with lower coefficients and significance levels.

Interestingly, climate networks that were originally founded to support mitigation action also significantly correlate with adaptation plans (apart from the Climate Alliance). Adaptation plans are mostly found among the cities of the C40 network and the CoM. The highest correlation coefficient is found between CoM membership and adaptation planning, highlighting the importance of this network in Europe. Climate networks have been criticized for their limited success in fostering climate action on the ground [76]. This criticism is not supported by the evidence of this sample, with the Covenant of Mayors being particularly successful in helping cities to prepare climate plans. It can be assumed that climate networks are important in raising awareness and increasing knowledge of climate change [77]. However, the fact that a climate plan exists does not provide evidence of its implementation or the success of climate actions [78,79]. Moreover, it is debateable whether climate networks are a driver or a consequence of climate planning. Membership of a climate network could be considered a form of climate action, taken by cities that are already environmentally active—although this might depend on the definition of climate action. Posey [53] showed that anticipated impacts can induce membership in climate networks (e.g. the CCP in the US) for cities at high risk from climate change—measured as coastal proximity, and expected temperature change and casualties of extreme weather events—suggesting that becoming a member of a climate network can be regarded a first step towards adaptation. Our analysis shows that joining a climate network may or may not be followed by additional action, i.e. the adoption of climate plans, but some climate networks appear to be very successful in helping cities to do so. Our analyses also show that it is mostly large and prosperous cities that engage in climate networks. This is likely explained by the institutional, economic or staff resources required in order to join the network [22], or by lobbying practices that make membership more difficult for smaller cities [43]. It is also noteworthy that some cities are members of multiple networks, which is presumably indicative of a general interest in climate issues and propensity to environmental behaviour [78].

Other socio-economic factors, such as population density and GDP per capita are also significant for adaptation planning. Our findings support previous studies that found that personal affluence is an influential driver of urban climate planning in the US [53]. However, the unemployment rate is not significant for adaptation in our sample, contrary to the case study there [53]. As mentioned before, this might be a consequence of the different planning contexts in Europe and the US.

Our study reveals a relatively large number of barriers to adaptation planning, compared with mitigation. A notable result is the significant negative correlation with the smart city ranking; although the number of cities for which this relationship can be assessed is small (*N* = 33), thereby increasing uncertainty. Smart cities in our sample, which invest in ICT to harness and develop their social and environmental capital, seldom have adaptation plans. It appears that these cities have either committed all their resources to the smart city concept and/or may see a conflict between being smart and being adaptive.

Being in the low elevation coastal zone and very close to the sea decreases the chance of having an adaptation plan. Being at risk of flooding from sea level rise is therefore not a driver of climate change plans—a noteworthy result. Being located by the sea might itself be a barrier to action in a wider sense, because adaptation to this and other locational factors demands considerable infrastructure and financial resources; or location could be a proxy variable for other barriers that affect coastal cities.

The same holds for cities with relatively warm summer temperatures. These cities, despite being at risk from higher temperatures and heat waves in the future, have fewer adaptation plans. This relates particularly to cities in Southern Europe as shown in Fig 1. Their lack of engagement with adaptation planning might be due to an underestimation of the risk or indicative of lower governance and coping capacity. This in turn could be connected to the fact that environmental taxes are lower in Southern European countries as compared with Northern ones [80], or simply be a consequence of lower GDP. Further explanations could include a lack of higher level government support or insufficient direction from the higher to the lower government levels [50,81], as well as lower perceived risk arising from extreme temperature compared with other environmental hazards [82–84].

The composite vulnerability factors underline these findings. Cities at risk of severe climate change impacts and with a high degree of future vulnerability (the latter indicator incorporates current adaptive capacity) currently have fewer adaptation plans. Moreover, Table 2 shows that the current adaptive capacity of a city is more strongly associated with the existence of adaptation plans than future impact and future vulnerability. We can therefore conclude that the current capacity of a city to engage in climate actions is a more important driver of adaptation planning than anticipated impacts and anticipated vulnerability. Indeed Hinkel [63] found that focusing on vulnerability indicators is not necessarily the right way to increase adaptive capacity or raise awareness of climate change.

Cities at risk from climate change, such as those in low-lying coastal areas and in hot climates, are not more likely to engage in climate planning; on the contrary, they engage less. Our analysis reveals that cities in high risk areas possess significantly less adaptive capacity—the most important factor for climate change adaptation planning. Adaptive capacity is most strongly associated with GDP per capita, which in turn is significantly lower in cities in high-risk areas. This comes with the caveat that barriers and drivers are interconnected; as highlighted by Engle [30], measures of vulnerability need to consider adaptive capacity.

A number of drivers and barriers identified in the literature as affecting adaptation plans are not significantly influential on the wider European level. These include: membership of the Climate Alliance—which is a network primarily focusing on mitigation; population density; the unemployment rate; median age of the population; height of the city above sea level; climatic attributes such as hours of sunshine per day, winter temperature, rain days per annum and amount of rainfall; proportion of green space and access to it; and mitigative capacity (see S3 Table). As already noted for mitigation, it is interesting that ‘green’ cities do not engage in climate change action more than ‘non-green’ cities. Climate change action seems therefore not necessarily motivated by a propensity for green or environmental behaviour. It is also notable that cities with a high proportion of elderly citizens—considered to be a particularly vulnerable strata of the population—are not especially motivated to engage in adaptation (or mitigation) planning. Here again the conclusion is that climate risk does not necessarily incentivize to proactive planning.

### Regression analyses with significant factors

Binary logistic regression tests whether a combination of chosen descriptors can predict whether a city has a mitigation or an adaptation plan. Here, we test a model with 6 predictors, including factors from each dimension (socio-economic, natural characteristics, and composite climate indices), that are significantly associated with climate plans and show relatively low collinearity. We exclude membership of climate networks, as joining a climate network can in itself be regarded as a climate action or response.

Using all factors that correlate highly with mitigation plans (*p*<0.001) yields a model of good fit (Hosmer and Lemeshow test is not significant (*p* = .883)) and significantly predicts whether a city has a mitigation plan (*χ* ^*2*^ = 54.14, *df* = 5, *N* = 101, *p* < .0001). The prediction is correct in 82% of the cases (82.9% correct where no mitigation plan exists; 81.7% correct where a mitigation plan is in place). Assessing the ROC curve yields an area under the curve of 0.89.

For mitigation, the population size, unemployment rate and adaptive capacity of a city are important factors with a significant contribution to the model. The odds of having a mitigation plan increase by 6% for every ten thousand inhabitants and by almost 19% for every unit of adaptive capacity (here calculated as an index between 0 and 100). The odds decrease by 26% (.743) for every percentage increase in the unemployment rate (Table 3). Although the unemployment rate is negatively correlated with mitigation planning, the direction of cause and effect is unclear, i.e. whether mitigation planning reduces unemployment, or unemployment hinders mitigation planning. The latter might be a consequence of the fact that mitigation planning would require the commitment of resources that are needed for other urban policies, e.g. job creation schemes. In light of the importance of (adaptive) capacity for mitigation planning it seems more plausible that a high unemployment rate reduces the availability of resources for climate action and hinders mitigation planning (rather than that mitigation planning creates employment).

**Table 3:** Binary regression results for mitigation plans.

	*ß* (B)	SE	Odds Ratio (Exp (B))	*p* of Wald statistic
Population size	.006	.002	1.006	.003
Population density	.000	.000	1.000	.230
GDP/ capita	.000	.000	1.000	.102
Unemployment rate	-.298	.093	.743	.001
Current Adaptive Capacity	.169	.048	1.185	.000
Constant	-7.669	2.531	.000	.002

For adaptation plans (Table 4), the population size of a city and its adaptive capacity are significant predictors. The model is a relatively good fit (Hosmer and Lemeshow test, *p* = .43) and can significantly predict whether a city has an adaptation plan (*χ* ^*2*^ = 23.71, *df* = 5, *N* = 153, *p* < .001). However, although overall 83% of all cases are predicted correctly, the absence of an adaption plan is correctly predicted with far greater frequency (97.6% correct) than the existence of one (only 17.9% correct). Assessing the ROC curve yields an area under the curve of 0.72.

**Table 4:** Binary regression results for adaptation plans.

	*ß* (B)	SE	Odds Ratio (Exp (B))	*p* of Wald statistic
Population size	.001	.000	1.001	.033
Coastal proximity	-2.048	1.162	.129	.078
Future CC impact	-.051	.032	.950	.111
Adaptive capacity	.110	.048	1.116	.022
Future CC vulnerability	.089	.054	1.093	.096
Constant	-9.652	3.668	.000	.009

The likelihood that a city has an adaptation plan increases by 1% with every ten thousand inhabitants and by 12% with every unit of adaptive capacity.

Despite the detection of highly significant factors for adaptation and mitigation planning in our sample, it is important to note that a number of the relations have rather low correlation coefficients. This means that either all tested factors have similarly low explanatory power or the explanatory power of factors is highly divergent across the sample and becomes rather small when averaged. However, testing potentially relevant alternative drivers and barriers suggested in the literature is difficult because of a lack of comparable data across political contexts, countries, and cities. Potential alternative drivers include political leadership [5,8,23], supportive political elites and political champions within cities, the presence of large powerful industries, engagement of partners from civil society, and the occurrence of a window of opportunity to connect climate action to large-scale, international (e.g. sporting) events [22]. Where similar data are available, definitions, and procedures for measurement and quantification are often incompatible across cities and countries [22]. Finally, in identifying significant barriers and drivers, this study has not considered the potential for interdependency between them.

Our results are informative for large-scale international comparison of the influence of factors identified as significant in the available literature. An advantage of the study is the inclusion of medium-sized cities [51]. However, correlation and regression analysis cannot determine causal relations. We therefore encourage the research community to conduct detailed analyses probing the associations identified in this study and to investigate the role of influences that are harder to quantify, for example by using mixed-method approaches [84,85] or qualitative analyses or interviews with decision makers [27], in order to develop broadly applicable indices incorporating the characteristics of cities most frequently aligned with climate change response. Having a climate change plan indicates awareness of the cross-sectoral challenges that climate change poses to the urban environment and signals a city’s general intent to engage in strategic climate actions. However, having a mitigation or adaptation plan is only one indicator and may not capture all activities within urban areas that are relevant to adaptation and mitigation [86], but signals a city’s general intent to engage in strategic climate actions. Our analysis shows that it is particularly difficult to predict adaptation plans. This suggests that adaptation plans are indeed more context specific than mitigation plans, as already noted in the literature [87], and/or more often developed on the regional level [4]. Future studies should also investigate the implementation process [46], and document the success of climate change plans [76]. Comparable research would also benefit from an improved categorization or typology of barriers and drivers of climate change plans and actions.

## Conclusion

Based on a selection of institutional, socio-economic and environmental factors, our study identifies drivers and barriers affecting urban climate change plans across 200 European cities through comparative and statistical analysis. Correlation and regression analyses reveal that institutional, socio-economic and environmental factors are all important as barriers and drivers of climate plans. Notable results include that climate networks are very effective support mechanisms for both mitigation and adaptation planning, that predominantly large and economically prosperous cities engage in mitigation and adaptation planning, and that particularly vulnerable cities—in terms of future climate risk and anticipated impacts—have significantly fewer mitigation and adaptation plans today. The current capacity of a city to engage with climate planning is thus more important than future anticipated impacts or vulnerability. This result highlights that climate planning, particularly for adaptation, is mostly reactive. Although policymakers have little power to alter environmental factors such as climatic variables or location, an understanding of how these factors are (or are not) related to engagement in climate planning is useful and necessary, for example, for the development of dedicated awareness campaigns and deciding where to target financial and institutional support. Table 5 lists the significant drivers and barriers of climate plans.

**Table 5:** Significant (*p*<0.05) drivers and barriers of climate change mitigation and adaptation plans across European cities. Drivers and barriers are listed in decreasing order of influence, i.e. from highest to lowest correlation coefficient.

	Significant drivers	Significant barriers
Mitigation plan	Covenant of Mayors member Population size GDP/ capita Climate AllianceCurrent Adaptive Capacity ICLEI member Population density C40 member	Unemployment rate Proximity to coast, i.e. <10km Average summer temperature Average winter temperature Future CC vulnerability
Adaptation plan	Covenant of Mayors member Current Adaptive Capacity Population sizeC40 member ICLEI member GDP/ capita Population density	Smart Cities rank Proximity to coast, i.e. <10kmFuture CC vulnerability Future CC impact Low elevation coastal zone Summer temperature

Regression analyses reveal that the odds of having a mitigation plan increase significantly with increases in a city’s population size and its adaptive capacity, while the odds decrease with a rise in the unemployment rate. The odds of having an adaptation plan also increase significantly in line with a city’s population size and its adaptive capacity. In light of the significance and importance of adaptive capacity for both adaptation and mitigation it seems likely that a high unemployment rate places demands on resources that are needed for other urban policies, e.g. job creation schemes, and thereby restricts the availability of funds for mitigation planning (rather than that mitigation planning induces employment). Overall, it is more difficult to predict which city has an adaptation plan than which city has a mitigation plan.

With respect to policy implications, we conclude that in particular smaller and economically weaker European cities, as well as those at high risk from future climate change, need support to engage in mitigation and adaptation. A dedicated climate network for highly vulnerable and potentially highly impacted cities, e.g. those at close proximity to the seas, might be very useful. We call for more specific, immediate and comprehensive economic and institutional support for smaller cities, cities with fewer resources and those potentially at risk of severe climate change impacts in the future. This group of cities would also be principal beneficiaries of higher-level programs and plans, whereby national policy acts as an institutional umbrella, guiding and supporting action by cities.

## Supporting Information

S1 Table Distribution of Urban Climate Change Plans across Countries. This table shows an overview of the existence of urban climate change plans across selected European countries. We list the total number of cities that have no plan, a mitigation plan, an adaptation plan, and an integrated adaptation and mitigation plan. We also point to climate leaders, which are cities with an adaptation plan, a mitigation plan, and quantitative mitigation targets in the mitigation plan.
(DOCX)

S2 Table List of surveyed cities and the names of available climate change plans. This table names the cities and the original titles of their planning/ policy documents reviewed alphabetically by country. All plans were published, authorized and drafted by the relevant local authority or the equivalent government office, or an authority or organization commissioned by the relevant local authority.
(DOCX)

S3 Table Results of correlation analyses. The following table shows the results of the correlation analysis in a matrix structure. It provides the correlation coefficient (*r*) together with the significance level (*p*-value) for all tested pairs.
(DOCX)

S1 Text Selection process and analysis of planning documents. The following text provides details on the selection process of urban climate change plans, as well as on their analysis. We describe the search mechanism, name search locations and list the keywords used. Additionally, we explain the form and way of content analysis conducted.
(DOCX)

S2 Text Forms and units of primary and secondary statistical data. The following list names the data with potential relevance to urban climate change policy and governance that we collected for analysis. We provide the name of data, their units, year of collection, and source of origin.
(DOCX)
