# Supplementary material for: The Influence of Drivers and Barriers on Urban Adaptation and Mitigation Plans—An Empirical Analysis of European Cities
Source: PLoS One. 2015 Aug 28;10(8):e0135597. doi: 10.1371/journal.pone.0135597 (PMC4552871; doi:10.1371/journal.pone.0135597)
Supplement: S1 Table — This table shows an overview of the existence of urban climate change plans across selected European countries. We list the total number of cities that have no plan, a mitigation plan, an adaptation plan, and an integrated adaptation and mitigation plan. We also point to climate leaders, which are cities with an adaptation plan, a mitigation plan, and quantitative mitigation targets in the mitigation plan. (DOCX) [file pone.0135597.s001.docx]

# S1 Table: Distribution of Urban Climate Change Plans across Countries. This table shows an overview of the existence of urban climate change plans across selected European countries. We list the total number of cities that have no plan, a mitigation plan, an adaptation plan, and an integrated adaptation and mitigation plan. We also point to climate leaders, which are cities with an adaptation plan, a mitigation plan, and quantitative mitigation targets in the mitigation plan.

| Urban Audit Cities | | | | | | | | | | | |
| --- | --- | --- | --- | --- | --- | --- | --- | --- | --- | --- | --- |
|  | Cities | ... with: | | | | | | | | Climate  leaders | |
|  |  | No plan | | Mitigation plan | | Adaptation plan | | Joint mitigation & adaptation plan | |  | |
| ...in country | N | N | [%] | N | [%] | N | [%] | N | [%] | N | [%] |
| Austria | 5 | 2 | 40.0 | 3 | 60.0 | 0 | 0.0 | 0 | 0.0 | 0 | 0.0 |
| Belgium | 7 | 4 | 57.1 | 3 | 42.3 | 0 | 0.0 | 0 | 0.0 | 0 | 0.0 |
| Estonia | 2 | 1 | 50.0 | 1 | 50.0 | 0 | 0.0 | 0 | 0.0 | 0 | 0.0 |
| Finland | 4 | 1 | 25.0 | 3 | 75.0 | 2 | 50.0 | 2 | 50.0 | 1 | 25.0 |
| France | 35 | 20 | 57.1 | 15 | 42.9 | 8 | 22.9 | 6 | 17.1 | 8 | 22.9 |
| Germany | 40 | 8 | 20.0 | 32 | 80.0 | 13 | 32.5 | 6 | 15.0 | 12 | 30.0 |
| Ireland | 4 | 2 | 50.0 | 2 | 50.0 | 0 | 0.0 | 0 | 0.0 | 0 | 0.0 |
| Italy | 32 | 14 | 43.8 | 18 | 56.3 | 1 | 3.1 | 0 | 0.0 | 1 | 3.1 |
| Netherlands | 15 | 3 | 20.0 | 12 | 80.0 | 3 | 20.0 | 2 | 13.3 | 3 | 20.0 |
| Spain | 26 | 13 | 50.0 | 13 | 50.0 | 5 | 19.2 | 3 | 11.5 | 4 | 15.4 |
| United Kingdom | 30 | 2 | 6.7 | 28 | 93.3 | 24 | 80.0 | 24 | 80.0 | 20 | 66.7 |
| TOTAL | 200 | 70 | 35.0 | 130 | 65.0 | 56 | 28.0 | 43 | 21.5 | 49 | 24.5 |
