# Supplementary material for: The Influence of Drivers and Barriers on Urban Adaptation and Mitigation Plans—An Empirical Analysis of European Cities
Source: PLoS One. 2015 Aug 28;10(8):e0135597. doi: 10.1371/journal.pone.0135597 (PMC4552871; doi:10.1371/journal.pone.0135597)
Supplement: S2 Table — This table names the cities and the original titles of their planning/ policy documents reviewed alphabetically by country. All plans were published, authorized and drafted by the relevant local authority or the equivalent government office, or an authority or organization commissioned by the relevant local authority. (DOCX) [file pone.0135597.s002.docx]

# S2 Table: List of surveyed cities and the names of available climate change plans. This table names the cities and the original titles of their planning/ policy documents reviewed alphabetically by country. All plans were published, authorized and drafted by the relevant local authority or the equivalent government office, or an authority or organization commissioned by the relevant local authority.

Key: Capital cities and cities of the C40 network are highlighted in **Capital**. n.a. - Not available; n.y. – no year.

| **City** | **Country** | **Mitigation strategy name** | **Adaptation strategy name** |
| --- | --- | --- | --- |
| Graz | Austria | KEK - Kommunales Energiekonzept (2011) | n.a. |
| Innsbruck | Austria | n.a. | n.a. |
| Linz | Austria | 50 (Plus-) Punkte für das Weltklima (2002) | n.a. |
| Salzburg | Austria | n.a. | n.a. |
| **Vienna** | **Austria** | **Klimaschutzprogramm Wien (2009)** | **n.a.** |
| Antwerpen | Belgium | Klimaatplan Antwerpen (2011) | n.a. |
| Brugge | Belgium | n.a. | n.a. |
| **Brussels** | **Belgium** | **Vers une Region Bruxelloise sobre en carbone a l'horizon 2025 (2010)** | **n.a.** |
| Charleroi | Belgium | n.a. | n.a. |
| Gent | Belgium | Lokaal Klimaatplan 2008 – 2020 (2008) | n.a. |
| Liège | Belgium | n.a. | n.a. |
| Namur | Belgium | n.a. | n.a. |
| **Tallinn** | **Estonia** | **Tallinna säästva energiamajanduse tegevuskava aastateks 2011-2021; "Energy savingsplan..." (2011)** | **n.a.** |
| Tartu | Estonia | n.a. | n.a. |
| **Helsinki** | **Finland** | **Capital Region Climate Strategy 2030 (2007)** | **n.a.** |
| Oulu | Finland | Oulu Region Climate Strategy (2009) | Oulu Region Climate Strategy (2009) |
| Tampere | Finland | Subregional climate change strategy 2030 (2010) | Subregional climate change strategy 2030 (2010) |
| Turku | Finland | n.a. | n.a. |
| Aix-en-Provence | France | n.a. | n.a. |
| Ajaccio | France | n.a. | n.a. |
| Amiens | France | n.a. | n.a. |
| Besançon | France | Plan Climat Energie du territoire du Grand Besançon (n.a.) | n.a. |
| Bordeaux | France | Plan Climat de la Cub (2011) | Plan Climat de la Cub (2011) |
| Caen | France | Plan Climat Energie Caen-Métropole: Diagnostic-Stratégie, un cadre d’actions pour agir (2010) | Etude sur les impacts, la vulnérabilité et l'adaptation du Calvados au changement climatique (2011) |
| Cayenne | France | n.a. | n.a. |
| Clermont-Ferrand | France | n.a. | n.a. |
| Dijon | France | ILLICO² - Plan Climat Energie du Grand Dijon (2010) | n.a. |
| Fort-de-France | France | n.a. | n.a. |
| Grenoble | France | Plan Climat Grenoble Alpes Métropole (2005) | Adaptation au changement climatique (2006) |
| Le Havre | France | n.a. | n.a. |
| Lens - Liévin | France | n.a. | n.a. |
| Lille | France | Plan Climat Energies Lille Métropole (n.a.) | n.a. |
| Limoges | France | n.a. | n.a. |
| Lyon | France | Agenda 21 Grand Lyon (2007) | Agenda 21 Grand Lyon (2007) |
| Marseille | France | n.a. | n.a. |
| Metz | France | n.a. | n.a. |
| Montpellier | France | n.a. | n.a. |
| Nancy | France | n.a. | n.a. |
| Nantes | France | Plan Climat Territorial Nantes Métropole (2007) | n.a. |
| Nice | France | n.a. | n.a. |
| Orléans | France | n.a. | n.a. |
| **Paris** | **France** | **Plan Climat de la ville de Paris (2007)** | **Plan Climat de la ville de Paris (2007)** |
| Pointe-á-Pitre | France | na | n.a. |
| Poitiers | France | Contrat Local Initiative Climat Grand Poitiers (2007) | n.a. |
| Reims | France | n.a. | n.a. |
| Rennes | France | Plan Climat Energie de la Ville de Rennes (2010) | n.a. |
| Rouen | France | n.a. | n.a. |
| Saint Denis | France | n.a. | n.a. |
| Saint-Etienne | France | Plan Climat Saint Etienne Métropole (2011) | Plan Climat Saint Etienne Métropole (2011) |
| Strasbourg | France | Plan Climat territorial de la Communauté urbaine de Strasbourg (2010) | n.a. |
| Toulon | France | n.a. | n.a. |
| Toulouse | France | Livre Blanc du Plan Climat (2012) | Livre Blanc du Plan Climat (2012) |
| Tours | France | Plan Climat 2011-2014 (2010) | Plan Climat 2011-2014 (2010) |
| Augsburg | Germany | CO2- Minderungskonzept für die Stadt Augsburg (2004) | n.a. |
| **Berlin** | **Germany** | **Energiekonzept 2020 (2011)** | **Stadtentwicklungsplan Klima (2011)** |
| Bielefeld | Germany | Handlungsprogramm Klimaschutz 2008-2020 (Klimaschutzteilkonzept) (2008) | Anpassungslösungen zur Milderung der Belastung für die überwärmten Stadtgebiete und zum Schutz der stadtnahen Waldgebiete (Klimaschutzteilkonzept) (2011) |
| Bochum | Germany | Energie- und Klimaschutzkonzept der Stadt Bochum für 2020 (2011) | n.a. |
| Bonn | Germany | Aktionsprogramm Klimaschutz (2007) | n.a. |
| Bremen | Germany | Klimaschutz- und Energieprogramm 2020 (2009) | n.a. |
| Darmstadt | Germany | n.a. | n.a. |
| Dortmund | Germany | Handlungsprogramm Klimaschutz 2020 (2011) | n.a. |
| Dresden | Germany | Integriertes Energie- und Klimaschutzkonzept (IEuKK) (2012) | Regionales Klimaanpassungsprogramm (IRKAP) (2012) |
| Düsseldorf | Germany | Klimaschutzprogramm (2008) | Klimaschutzprogramm (2008) |
| Erfurt | Germany | n.a. | n.a. |
| Essen | Germany | Integriertes Energie- und Klimakonzept der Stadt Essen (2009) | Integriertes Energie- und Klimakonzept der Stadt Essen (2009) |
| Frankfurt am Main | Germany | Energie- und Klimaschutzkonzept der Stadt Frankfurt am Main (2008) | n.a. |
| Frankfurt an der Oder | Germany | n.a. | n.a. |
| Freiburg im Breisgau | Germany | Klimaschutzstrategie der Stadt Freiburg (2007) | n.a. |
| Göttingen | Germany | Integriertes Klimaschutzkonzept für das Stadtgebiet Göttingen (2010) | n.a. |
| Halle an der Saale | Germany | n.a. | n.a. |
| Hamburg | Germany | Hamburger Klimaschutzkonzept (2011) | Hamburger Klimaschutzkonzept (2011) |
| Hannover | Germany | Klima-Allianz Hannover 2020 (2008) | n.a. |
| Karlsruhe | Germany | Klimaschutzkonzept Karlsruhe (2009) | Anpassung an den Klimawandel (2012) |
| Kiel | Germany | Kieler Energie- und Klimaschutzkonzept (2008) | n.a. |
| Koblenz | Germany | Integriertes Klimaschutzkonzept für die Stadt Koblenz (2011) | n.a. |
| Köln | Germany | Klimaschutzkonzept Köln – vorgezogenes Sofortmaß-nahmenprogramm (2011) | Klimawandelgerechte Metropole Köln - Strategien zur Anpassung an den Klimawandel (2012) |
| Leipzig | Germany | Energie- und Klimaschutzkonzept (2011) | n.a. |
| Magdeburg | Germany | n.a. | n.a. |
| Mainz | Germany | Energiekonzept Mainz 2005-2015 – Energie und Verkehr (2008) | n.a. |
| Moers | Germany | Integriertes kommunales Klimaschutzkonzept, Stadt Moers (2011) | Flächenbericht 2009 - Stadt Moers (2010) |
| Mönchenglad-bach | Germany | Integriertes kommunles Klimaschutzkonzept (2011) | n.a. |
| Mülheim an der Ruhr | Germany | Klimaschutzkonzept (2010) | Handlungsansätze und Leitgedanken zu Klimaschutz und Klimaanpassung (2011) |
| München | Germany | Integriertes Handlungsprogramm Klimaschutz München (2010) | n.a. |
| Nürnberg | Germany | Klimaschutzfahrplan 2010/2020 (2010) | "Handbuch Klimaanpassung - Bausteine für die Nürnberger Anpassungsstrategie" (2012) |
| Potsdam | Germany | Integriertes Klimaschutzkonzept, (2010) | Integriertes Klimaschutzkonzept (2010) |
| Regensburg | Germany | n.a. | n.a. |
| Rostock | Germany | Klimaschutz - Ein Rahmenkonzept für die Hansestadt Rostock; 1. Fortschreibung 2010 – 2020 (2010) | n.a. |
| Saarbrücken | Germany | n.a. | n.a. |
| Schwerin | Germany | n.a. | n.a. |
| Stuttgart | Germany | Fortschreibung des Klimaschutzkonzepts (2007) | Klimaanpassungskonzept Stuttgart KLIMAKS (2012) |
| Trier | Germany | Integriertes Klimaschutzkonzept der Region Trier (2011) | n.a. |
| Weimar | Germany | Integriertes Klimaschutzkonzept "Strom, Wärme, Kälte" (2011) | n.a. |
| Wiesbaden | Germany | Energiekonzept Wiesbaden 2020 (2011) | n.a. |
| Cork | Ireland | Cork City Council Climate Change Strategy (2010) | n.a. |
| Dublin | Ireland | Climate Change Strategy for Dublin City (2008) | n.a. |
| Galway | Ireland | n.a. | n.a. |
| Limerick | Ireland | n.a. | n.a. |
| Ancona | Italy | n.a. | n.a. |
| Bari | Italy | Piano di Azione per l'Energia Sostenibile (2011) | n.a. |
| Bologna | Italy | Programma Energetico Comunale (2007) | n.a. |
| Brescia | Italy | Piano Energetico Comunale (2002) | n.a. |
| Cagliari | Italy | n.a. | n.a. |
| Campobasso | Italy | n.a. | n.a. |
| Caserta | Italy | n.a. | n.a. |
| Catania | Italy | n.a. | n.a. |
| Catanzaro | Italy | n.a. | n.a. |
| Cremona | Italy | n.a. | n.a. |
| Firenze | Italy | Piano di Azione per l'Energia Sostenibile del Comune di Firenze (2011) | n.a. |
| Foggia | Italy | Piano Energetico Comunale (2007) | n.a. |
| Genova | Italy | Piano d'Azione per l’energia sostenibile (2010) | n.a. |
| L'Aquila | Italy | n.a. | n.a. |
| **Milan** | **Italy** | **Piano d'Azione per l’energia sostenibile e il clima (2009)** | **n.a.** |
| Modena | Italy | Piano d'Azione per l’energia sostenibile (2011) | n.a. |
| Napoli | Italy | Piano d’Azione 20-20-20 (2008) | n.a. |
| Padova | Italy | Piano d'Azione per l’energia sostenibile (2011) | Piano Clima (n.y.) |
| Palermo | Italy | Piano Energetico Comunale (2000) | n.a. |
| Perugia | Italy | Piano Energetico Ambientale Comunale (2005) | n.a. |
| Pescara | Italy | n.a. | n.a. |
| Potenza | Italy | n.a. | n.a. |
| Reggio di Calabria | Italy | n.a. | n.a. |
| **Rome** | **Italy** | **Piano d'Azione per Kyoto (2004)** | **n.a.** |
| Salerno | Italy | Proposta di Piano Energetico Comunale (2009) | n.a. |
| Sassari | Italy | n.a. | n.a. |
| Taranto | Italy | n.a. | n.a. |
| Torino | Italy | Piano d'Azione per l'Energia Sostenibile (2010) | n.a. |
| Trento | Italy | Piano Energetico Ambientale del Comune di Trento: Trento per Kyoto (2007) | n.a. |
| Trieste | Italy | n.a. | n.a. |
| Venezia | Italy | Piano Energetico Comunale (2009) | n.a. |
| Verona | Italy | Proposta di Piano d’Azione per l’Energia Sostenibile (2010) | n.a. |
| Alicante/  Alacant | Spain | n.a. | n.a. |
| Badajoz | Spain | n.a. | n.a. |
| **Barcelona** | **Spain** | **PECQ - Pla d l'Energia, el Canvi Climàtic i la Qualitat de l'Aire de la ciutat de Barcelona (2011)** | **n.a.** |
| Bilbao | Spain | Plan Local de Acción contra el Cambio Climático de Bilbao (2010) | n.a. |
| Córdoba | Spain | n.a. | n.a. |
| Coruña, A | Spain | n.a. | n.a. |
| Gijón | Spain | n.a. | n.a. |
| Las Palmas | Spain | n.a. | n.a. |
| L'Hospitalet de Llobregat | Spain | Pla d'Acci'o per l'Energia Sostenible (n.y.) | n.a. |
| Logroño | Spain | Plan Local para la lucha contra el cambio climático (n.y.) | n.a. |
| **Madrid** | **Spain** | **Plan de Uso Sostenible de la Energía y Prevención del Cambio Climático de la Ciudad de Madrid (2008)** | **Plan de Uso Sostenible de la Energía y Prevención del Cambio Climático de la Ciudad de Madrid (2008)** |
| Málaga | Spain | Plan de Acción para la Energía Sostenible de Málaga (2010) | n.a. |
| Murcia | Spain | Estrategia Local frente al cambio climático del Municipio de Murcia (2008) | Estrategia Local frente al cambio climático del Municipio de Murcia (2008) |
| Oviedo | Spain | n.a. | n.a. |
| Palma di Mallorca | Spain | n.a. | n.a. |
| Pamplona/ Iruña | Spain | Plan de Acción para la Energía Sostenible de Pamplona (n.y.) | n.a. |
| Santa Cruz de Tenerife | Spain | n.a. | n.a. |
| Santander | Spain | n.a. | n.a. |
| Santiago de Compostela | Spain | n.a. | n.a. |
| Sevilla | Spain | Plan de Acción para la Energía Sostenible de Sevilla (2010) | n.a. |
| Toledo | Spain | n.a. | n.a. |
| Valencia | Spain | Plan de Acción para la Energía Sostenible de Valencia (2010) | Plan de Acción para la Energía Sostenible de Valencia (2010) |
| Valladolid | Spain | Plan de Actuación de la Agencia Energética Municipal de Valladolid (2002) | n.a. |
| Vigo | Spain | n.a. | n.a. |
| Vitoria/ Gasteiz | Spain | Plan de Lucha Contra el Cambio Climático 2010-2020 (2010) | Plan de Adaptación al Cambio Climático de Vitoria-Gasteiz (2012) |
| Zaragoza | Spain | Estrategia de Cambio Climático y Calidad del Aire de Zaragoza (2009) | Estrategia de Adaptación al Cambio Climático en la ciudad de Zaragoza (2010) |
| Almere | The Netherlands | n.a. | n.a. |
| **Amsterdam** | **The Netherlands** | **Energiestrategie Amsterdam 2040 Brug naar een duurzame energievoorziening (2010)** | **n.a.** |
| Apeldoorn | The Netherlands | n.a. | n.a. |
| Arnhem | The Netherlands | Arnhems Klimaatprogramma 2008-2011 (2009) | n.a. |
| Breda | The Netherlands | Steek positieve energie in het klimaat Breda: een CO2- neutrale stad in 2044 (2008) | n.a. |
| Eindhoven | The Netherlands | n.a. | n.a. |
| Enschede | The Netherlands | Nieuwe Energie voor Enschede “Versnellen en verscherpen van klimaataanpak door energie (2010) | n.a. |
| Gravenhage | The Netherlands | Klimaatplan Den Haag (2009) | n.a. |
| Groningen | The Netherlands | Routekaart Groningen Energieneutraal+ 2025 (2007) | n.a. |
| Heerlen | The Netherlands | Klimaatbeleidsplan Heerlen 2010-2020 (n.y.) | n.a. |
| Leeuwarden | The Netherlands | Klimaattop Leeuwarden - Slimme Energie-en Waterketens Tussen Mensen en Plaatselijke Bronnen (2010) | n.a. |
| Nijmegen | The Netherlands | Actieplan Klimaat 2008-2012 (2008) | Actieplan Klimaat 2008-2012 (2008) |
| **Rotterdam** | **The Netherlands** | **Mitigation Action Programme (2010)** | **Adaptation Programme 2010 Rotterdam Climate Proof (2010)** |
| Tilburg | The Netherlands | Eerste Klimaatprogramma Tilburg naar een Klimaatneutrale en Klimaatbestendige Stad (2009) | Eerste Klimaatprogramma Tilburg naar een Klimaatneutrale en Klimaatbestendige Stad (2009) |
| Utrecht | The Netherlands | Programma Utrechtse Energie 2011-2014 (2011) | n.a. |
| Aberdeen | UK | Climate Change Action Programme (2002) | Climate Change Action Programme (2002) |
| Belfast | UK | Sustainable Development Action Plan 2009-11 (2009) | Sustainable Development Action Plan 2009-11 (2009) |
| Birmingham | UK | Climate Change Action Plan 2010+ (2010) | Climate Change Action Plan 2010+ (2010) |
| Bradford | UK | Climate change strategy for Bradford District-Draft (2011) | Climate change strategy for Bradford District-Draft (2011) |
| Bristol | UK | Climate Change and Energy Security Framework (2010) | n.a. |
| Cambridge | UK | Climate Change Strategy and Action Plan 2008-2012 Final Draft (2008) | Climate Change Strategy and Action Plan 2008-2012 Final Draft (2008) |
| Cardiff | UK | Carbon Lite Action Plan (2010) | Carbon Lite Action Plan (2010) |
| Coventry | UK | Climate Change Strategy for Coventry (2008) | Climate Change Strategy for Coventry (2008) |
| Derry | UK | n.a. | n.a. |
| Edinburgh | UK | Sustainable Edinburgh 2020 (2011) | Sustainable Edinburgh 2020 (2011) |
| Exeter | UK | Climate Change Strategy 2008-18 (2008) | Climate Change Strategy 2008-18 (2008) |
| Glasgow | UK | Sustainable Glasgow Report 2010 (2010) | Sustainable Glasgow Report (2010) |
| Gravesham | UK | Climate Change and Environmental Protection –Baseline (2009) | n.a. |
| Kingston-upon-Hull | UK | Climate Change 2010-20 A low carbon framework (2010) | Climate Change 2010-20 A low carbon framework (2010) |
| Leeds | UK | Climate Change Strategy-Vision for Action (2010) | Climate Change Strategy-Vision for Action (2010) |
| Leicester | UK | Climate Change Mitigation Plan for Leicester (2010) | Climate Change Adaptation Plan for Leicester (2010) |
| Lincoln | UK | Climate Change Strategy Phase 1 (2005) | Climate Change Strategy Phase 1 (2005) |
| Liverpool | UK | Climate Change Strategic Framework: A prospectus of action (2009) | Climate Change Strategic Framework: A prospectus of action (2009) |
| **London** | **UK** | **Delivering London's Energy Future; The Mayor's CC Mitigation and Energy Strategy (2011)** | **Managing risks and increasing resilience; The Mayor’s climate change adaptation strategy (2011)** |
| Manchester | UK | Manchester -a certain future- CC action plan (2009) | Manchester -a certain future- CC action plan (2009) |
| Newcastle u. Tyne | UK | Citywide Climate Change Strategy & Action Plan 2010 – 2020 (2010) | Citywide Climate Change Strategy & Action Plan 2010 – 2020 (2010) |
| Nottingham | UK | Draft Community CC strategy (2011) | Draft Community CC strategy (2011) |
| Portsmouth | UK | Climate Change Strategy (2009) | Climate Change Strategy (2009) |
| Sheffield | UK | Carbon Reduction Framework (2009) | n.a. |
| Stevenage | UK | Climate Change Strategy (2009) | n.a. |
| Stoke-on-Trent | UK | Draft Sustainability and CC (2010) | Draft Sustainability and CC (2010) |
| Wirral | UK | Wirral Climate Change Strategy (2007) | Wirral Climate Change Strategy (2007) |
| Wolverhampton | UK | Climate Change Strategy and Action Plan 2009-12 (2009) | Climate Change Strategy and Action Plan 2009-12 (2009) |
| Worcester | UK | Worcester City Council Climate Change strategy (2009) | Worcester City Council Climate Change strategy (2009) |
| Wrexham | UK | n.a. | n.a. |
