# Supplementary material for: The Influence of Drivers and Barriers on Urban Adaptation and Mitigation Plans—An Empirical Analysis of European Cities
Source: PLoS One. 2015 Aug 28;10(8):e0135597. doi: 10.1371/journal.pone.0135597 (PMC4552871; doi:10.1371/journal.pone.0135597)

# S3 Table: Results of correlation analyses. The following table shows the results of the correlation analysis in a matrix structure. It provides the correlation coefficient (*r*) together with the significance level (*p*-value) for all tested pairs.


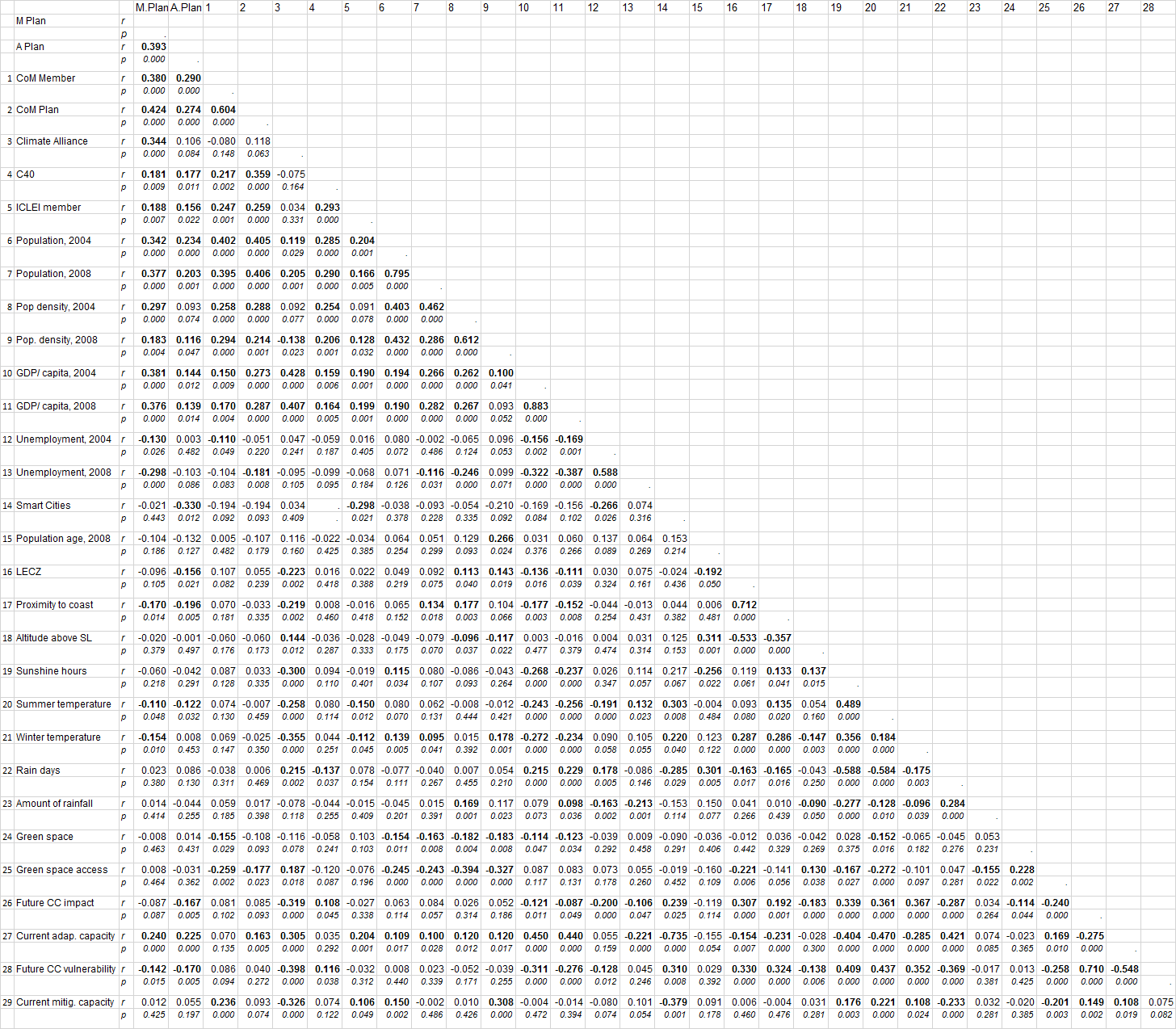

Supplement: S3 Table — The following table shows the results of the correlation analysis in a matrix structure. It provides the correlation coefficient (r) together with the significance level (p-value) for all tested pairs. (DOCX) [file pone.0135597.s003.docx]
