# Supplementary material for: The Influence of Drivers and Barriers on Urban Adaptation and Mitigation Plans—An Empirical Analysis of European Cities
Source: PLoS One. 2015 Aug 28;10(8):e0135597. doi: 10.1371/journal.pone.0135597 (PMC4552871; doi:10.1371/journal.pone.0135597)
Supplement: S1 Text — The following text provides details on the selection process of urban climate change plans, as well as on their analysis. We describe the search mechanism, name search locations and list the keywords used. Additionally, we explain the form and way of content analysis conducted. (DOCX) [file pone.0135597.s004.docx]

# S1 Text: Selection process and analysis of planning documents. The following text provides details on the selection process of urban climate change plans, as well as their analysis. We describe the search mechanism, name search locations and list the keywords used. Additionally, we explain the form and way of content analysis conducted.

Policy documents of all cities were obtained from local government websites through internet search or retrieved from planning offices or the city administration through direct contact. For the internet search following steps were followed:

- 1. Search in a common search engine with following keywords: [city name] Klimaschutz, Klimaschutzkonzept, Integriertes Klimaschutzkonzept, Anpassung an den Klimawandel (in the German case), or [city name] cambio climático, plan mitigación, plan mitigación cambio climático, adaptación cambio climático, plan adaptación cambio climático, ayuntamiento cambio climático, ayuntamiento medioambiente (in the Spanish case) and similar in the other respective languages;
  2. Search on the website of the city authority, in particular their environment, sustainability, energy and/or climate departments (or equivalent) of the local authority;

**Content analysis of planning documents**

The documents were analysed concerning their targets and foci. A scoping study focused on German and Austrian documents to derive an initial code for document analysis using Grounded Theory (Glaser and Strauss 1967). Grounded Theory is a systematic methodology deriving theory through the analysis of data (e.g. a text). Grounded theory starts with data collection and its analysis by which a theory or categorization of a subject is derived, rather than beginning with a hypothesis. In an iterative process the code was further developed and completed while analysing additional documents under the contribution of lead partners in the team.
