# Supplementary material for: The Influence of Drivers and Barriers on Urban Adaptation and Mitigation Plans—An Empirical Analysis of European Cities
Source: PLoS One. 2015 Aug 28;10(8):e0135597. doi: 10.1371/journal.pone.0135597 (PMC4552871; doi:10.1371/journal.pone.0135597)
Supplement: S2 Text — The following list names the data with potential relevance to urban climate change policy and governance that we collected for analysis. We provide the name of data, their units, year of collection, and source of origin. (DOCX) [file pone.0135597.s005.docx]

# S2 Text: Forms and units of primary and secondary statistical data. The following list names the data with potential relevance to urban climate change policy and governance that we collected for analysis. We provide the name of data, their units, year of collection, and source of origin.

Key: Information in brackets denotes units; abbreviations: cap - capita, km² - square kilometers, m² - square meters, # - number):

Dependent variables - Climate change plans:

1. Existence of a mitigation plan [yes/ no]
2. Existence of an adaptation plan [yes/ no]

Independent variables - Institutional factors:

1. Member of Covenant of Mayors [yes/ no], 01/2012 ^3^
2. Covenant of Mayors: Plan submitted [yes/ no], 01/2012 ^3^
3. Member of Climate Alliance [yes/ no], 01/2012 ^1^
4. Member of C40 member [yes/ no], 01/2012 ^2^
5. Member of ICLEI member [yes/ no], 01/2012 ^4^

Independent variables - Socio-economic factors:

1. Population [#], 2004 ^6^
2. Population [#], 2008 ^6^
3. Population density [cap/km²], 2004 ^6^
4. Population density [cap/km²], 2008 ^6^
5. GDP/ head [€], 2004 ^6^
6. GDP/ head [€],2008 ^6^
7. Unemployment rate [%], 2004 ^6^
8. Unemployment rate [%], 2008 ^6^
9. Smart Cities Index [0-100], 01/2012 ^5^
10. Median population age, 2008 ^6^

Independent variables - Environmental factors:

1. Low elevation coastal zone [yes/ no] ^7^
2. Proximity to coast <= 10 km [yes/ no] ^7^
3. Median city center altitude above sea level [m], 2006 ^6^
4. Total number of hours of sunshine per day [#], 2004/08 ^6^
5. Average temperature of warmest month [°C], 2004/08 ^6^
6. Average temperature of coldest month [°C], 2004/08 ^6^
7. Number of days of rain per annum [#], 2004/08 ^6^
8. Rainfall [liter/m2], 2004/08 ^6^
9. Proportion of green space area [% of city area], 2004/08 ^6^
10. Relative green space to which the public has access [m², per capita] , 2004/08 ^6^

Independent variables – ESPON Climate Indices:

1. Aggregated Impact [index], 2071-2100 ^8^
2. Combined Adaptive Capacity [index], 2005-2011 ^8^
3. Aggregated Vulnerability [index], 2071-2100 ^8^
4. Combined Mitigative Capacity [index], 2005-2011 ^8^

Additional, data from third-party is taken from the publicly accessible websites and sources:

^1^ - <http://www.klimabuendnis.org/home.html?&L=0>; [Access: 30.01.2013]

^2^ - <http://www.c40cities.org/>; [Access: 30.01.2013]

^3^ - <http://www.eumayors.eu/index_en.html>; [Access: 30.01.2013]

^4^ - <http://www.iclei.org/index.php?id=11454>; [Access: 29.01.2013]

^5^ - <http://www.smart-cities.eu>; [Access: 29.04.2013]

^6^ – Data for core cities from Eurostat, <http://epp.eurostat.ec.europa.eu/portal/page/portal/region_cities/city_urban/data_cities/database_sub1>; [Access: 31.01.2013]

^7^ – <http://maps.google.com>; [Access: 30.01.2013]; Self-evaluation with Google Maps Inc.

^8^ – <http://database.espon.eu/db2/home>; © ESPON Database, [Access: 26.11.2013]. Origin of the data: “ESPON Climate - Climate Change and Territorial Effects on Regions and Local Economies in Europe”, the Institute of Spatial Planning (IRPUD) of the Technical University Dortmund, Faculty of Spatial Planning (2011). The data are freely available at <http://database.espon.eu/db2/home>, but use is restricted to non-profit purposes under conditions of correct citation, see <http://database.espon.eu/db2/terms;jsessionid=4cc958de3afcc88ee77650855453>. Requests for data access can also be sent to: [irpud.rp@tu-dortmund.de](mailto:irpud.rp@tu-dortmund.de) or to [antoine.laporte@ums-riate.fr](mailto:antoine.laporte@ums-riate.fr).”
